# A different point of view: the evaluation of motor imagery perspectives in patients with sensorimotor impairments in a longitudinal study

**DOI:** 10.1186/s12883-021-02266-w

**Published:** 2021-07-27

**Authors:** Szabina Gäumann, Rahel Sarah Gerber, Zorica Suica, Jasmin Wandel, Corina Schuster-Amft

**Affiliations:** 1 Institute of Physiotherapy, School of Health Professions, Zurich University of Applied Sciences, Katharina-Sulzer-Platz 9, 8401 Winterthur, Switzerland; 2 Research Department, Reha Rheinfelden, Salinenstrasse 98, 4310 Rheinfelden, Switzerland; 3 Institute for Optimisation and Data Analysis, Bern University of Applied Sciences, Jlcoweg 1, 3400 Burgdorf, Switzerland; 4 Institute for Rehabilitation and Performance Technology, Bern University of Applied Sciences, Pestalozzistrasse 20, 3400 Burgdorf, Switzerland; 5 Division for Rehabilitative and Regenerative Sports Medicine, Department of Sport, Exercise and Health, University of Basel, Mittlere Allee 18, 4052 Basel, Switzerland

**Keywords:** Motor imagery, Motor imagery perspective, Neurorehabilitation, Sensorimotor impairment

## Abstract

**Background:**

Motor imagery (MI) has been successfully applied in neurological rehabilitation. Little is known about the spontaneous selection of the MI perspectives in patients with sensorimotor impairments. What perspective is selected: internal (first-person view), or external (third-person view)? The aim was to evaluate the MI perspective preference in patients with sensorimotor impairments.

**Methods:**

In a longitudinal study including four measurement sessions, 55 patients (25 stroke, 25 multiple sclerosis, 5 Parkinson’s disease; 25 females; mean age 58 ± 14 years) were included. MI ability and perspective preference in both visual and kinaesthetic imagery modalities were assessed using the Kinaesthetic and Visual Imagery Questionnaire-20 (KVIQ-20), the body rotation task (BRT), and mental chronometry (MC). Additionally, patients’ activity level was assessed. Descriptive analyses were performed regarding different age- (< 45, 45–64, > 64), activity levels (inactive, partially active, active), and KVIQ-20 movement classifications (axial, proximal, distal, upper and lower limb). A mixed-effects model was used to investiage the relationship between the primary outcome (MI perspective: internal, external) with the explanatory variables age, MI modality (visual, kinaesthetic), movement type (axial, proximal, distal), activity levels and the different assessments (KVIQ-20, BRT, MC).

**Results:**

Imagery modality was not a significant predictor of perspective preference. Over the four measurement sessions, patients tended to become more consistent in their perspective selection, however, time point was not a significant predictor. Movement type was a significant predictor: imagination of distal vs. axial and proximal vs. axial movements were both associated with preference for external perspective. Patients with increased physical activity level tend to use internal imagery, however, this effect was borderline not statistically significant. Age was neither a significant precictor. Regarding the MI assessments, the KVIQ- 20 score was a significant predictor. The patients with higher test scores tend to use the external perspective.

**Conclusion:**

It is recommended to evaluate the spontaneous MI perspective selection to design patient-specific MI training interventions. Distal movements (foot, finger) may be an indicator when evaluating the consistency of the MI perspective in patients with sensorimotor impairments.

**Supplementary Information:**

The online version contains supplementary material available at 10.1186/s12883-021-02266-w.

## Background

The active imagination of movements also called motor imagery (MI) is defined as the mental representation of an action without engaging in its actual execution [1]. In sport psychology, studies frequently reported that MI practice has a positive effect on motor performance [2, 3]. Over the last decades, MI has been successfully introduced to a wide range of disciplines, e.g. in education, medicine, and music [4]. In addition, the MI technique has been successfully integrated in the field of rehabilitation as an essential motor learning approach facilitating positive neurophysiological changes in the central nervous system and therefore, brain plasticity [1, 5]. Neuroimaging studies using positron emission tomography and functional magnetic resonance imaging (fMRI) demonstrated that physical execution and mental practice of a movement show similar brain activation pattern [6–8]. In neurological rehabilitation, MI practice seems to be an effective complementary therapy for patients with sensorimotor impairments providing additional benefits if added to conventional therapies [9–12]. Patients seem to benefit from MI practice because it can be safely practiced alone, thus expanding the time spent with rehabilitation-related activities [13].

There are two different perspectives described to imagine a movement: a) an internal and b) an external perspective. The internal view describes the first-person view, where the individual visualises the movement through his or her own view, comparable to what you would see through a camera mounted on the head. The external perspective describes the third-person view, where the individual watches him or herself performing an action from a spectator’s position [14, 15].

So far, preference was given to the internal perspective because it is believed to be more effective than external perspective MI practice. Both internal and external perspective imagery shows similar brain activation pattern. The activated brain areas were the supplementary motor area, the premotor cortex, the precentral gyrus, the lingual gyrus, the posterior cingulate cortex, the superior temporal lobe, the supramarginal gyrus, the precuneus and putamen [16, 17]. However, using fMRI technology, Lorey, et al. observed that the internal perspective leads to a stronger activation of motor and motor-related areas than the external perspective, especially in the inferior parietal lobe in the left hemisphere [17]. Regarding the muscular activity during MI, Harris and Robinson, and Bakker et al. described that internal imagery resulted a significant increase compared to external imagery during the imagination of weight lifting and arm lifts in students [18, 19]. Several studies have investigated the effectiveness of MI practice using internal perspective imagery for rehabilitation purposes [20–22]. Furthermore, in a review on MI training elements, Schuster, et al. reported, that in MI interventions with positive results, the internal perspective was more frequently used [4]. Also, the Kinaesthetic and Visual Imagery Questionnaire-20, which is standardized questionnaire to assess MI ability of patients with sensorimotor impairments, uses the first person perspective [23].

Only few studies investigated the explicit effects of MI perspectives on motor performance [14, 21, 22]. However, the available findings on MI perspective do not support the notion that the use of an internal perspective is consistently superior to an external perspective [24, 25]. In a sport context, White and Hardy [24] demonstrated that different MI perspectives could enhance different aspects of motor performance. Using an external perspective was found to be more effective for learning and retention of a task and improvement of speed, whereas using an internal perspective supported the accuracy of a performance [24]. Furthermore, evidence suggests that athletes rather take on an external perspective in open sports with complex movements [25, 26]. So far, the basis of the recommendation to select the internal or the external perspective is inconclusive.

Less is known about how patients with sensorimotor impairments imagine movements. Studies on MI involving patients focused on MI ability and reported less details concerning the MI perspectives. Randhawa et al. described that patients with Parkinson’s disease showed some difficulty to imagine axial movements (e.g. neck flexion) [27]. Furthermore, Dettmers et al. demonstrated better MI ability for the external perspective compared to the internal perspective in patients after a stroke [28]. Reports on patients’ MI perspective preferences were embedded in two further studies. Schuster et al. [29] and Wondrusch and Schuster-Amft [30] described that the majority of the included patients with sensorimotor impairments preferred an external MI perspective for imagining gestures, when they could freely choose a MI perspective.

Several factors that seem to influence the choice of a certain MI perspective were described in the literature. Mulder et al. [31] pointed out the importance of age. Their results indicated that healthy elderly participants (> 64 years) showed a reduced MI ability to imagine movements when using an internal perspective. The authors suggested to assess the MI ability of a participant before starting a MI training program. Furthermore, an age related shift to the external perspective preference was supported by Kalicinski et al. [32]. Indeed, when MI capacity decreases with age, the therapeutic application of MI practice in motor rehabilitation becomes uncertain, especially with regard to neurorehabilitation, as the majority of patients belong to the older population [33]. Furthermore, Mulder and colleagues discussed a possible association between the physical activity level and MI ability [31]. It was proposed that a decreased physical activity level during aging could have a negative influence on the ability to imagine movements, particularly to use the internal perspective when imagining movements [31].

There are further indications that healthy individuals as well as patients with sensorimotor impairments might change their MI perspective preference. Jiang et al. [16] reported that four out of 15 healthy participants aged between 19 and 29 years could not maintain their imagery perspective during brain scanning while imaging running up stairs. Seiler et al. [34] removed two participants from their study because they were switching MI perspectives. White and Hardy [24] had to exclude three out of 24 first-year sport health and physical education students from their analysis due to the same reason. A change of the preferred MI perspective in patients with motor impairments between two measurement sessions was further observed by Schuster et al. [29] when seven out of 73 patients changed their preferred MI perspective.

Apart from the MI perspective, the MI user may also choose between a visual and a kinaesthetic modality. The visual modality focuses on the visualisation of the movement, while the kinaesthetic modality focuses on the sensation of the movement [15]. The kinaesthetic modality is commonly allocated to the formation of an internal perspective. Further, Dijkerman et al. [13] claimed that only an internal perspective could accompany kinaesthetic imagery. Additionally, Jeannerod [35] proposed that an internal perspective involves kinaesthetic representations, while the external perspective refers to visual representation of an action. However, White and Hardy [24] and Callow et al. [14] suggested, that it might also be possible to experience a kinaesthetic modality while using an external perspective. In both studies with sport students and healthy young adults, both study groups used either an internal or an external perspective and reported equally kinaesthetic sensations during MI practice [14, 24].

Unfortunately, the perspective and the modality are not always recognised as distinct elements of the MI technique. While several studies provided information on how participants were instructed to engage in MI practice, most of studies did not disclose the modality used by the participants [36, 37]. Conversely, other studies make the distinction between modality but do not report the perspective [7, 15]. Meanwhile there are investigations exploring brain activations ivolving modalities and perspectives [16, 34]. They convey the importance of individual differences in the different conditions.

There seems to be contradictory evidence and it remains uncertain what might be the best combination of perspectives and modalities. To include MI in clinical practice and to develop useful instructions for MI training, it is important to understand the role of the perspectives in a clinical setting.

### Aim and hypothesis

The main aim of this study was to explore the spontaneous perspective selection over four measurement sessions in patients with sensorimotor impairments during visual and kinaesthetic MI practice and to investigate the realitonship between their preferred perspective and age, activity level, imagined movement types. A further interest was to examine the relationship between MI ability and perspective preference.

It was hypothesised that imagery modalities do not have an effect on perspective preference: patients select an internal or external perspective during both visual and kinaesthetic modalities, and that there might be changes of the perspective preference over time.

Furthermore, it was assumed that (1) age might have an effect on the preferred perspective and patients > 64 years would prefer an external perspective spontaneously, (2) the physical activity level might have an effect on the preferred perspective and patients being less active would prefer an external perspective spontaneously, (3) the imagined movement type might have an effect on the preferred perspective, and (4) MI ability might have an effect on the preferred perspective.

## Methods

### Participants

In- and outpatients were recruited in a neurorehabilitation clinic in the Northwestern part of Switzerland. Potential candidates received oral and written study information and were given at least 24 hours to consider participation. Written informed consent was obtained before data collection started. Patients were eligible for participation if they were older than 18 years, had a first-ever clinically confirmed stroke (PwSTR), or multiple sclerosis (PwMS), or Parkinson’s disease (PwPD), had a minimum score of 19 on the Montreal Cognitive Assessment (MoCA), were able to sit stable on an armless chair, and could read and understand German. Patients with persistent pain were excluded.

**Fig. 1:**
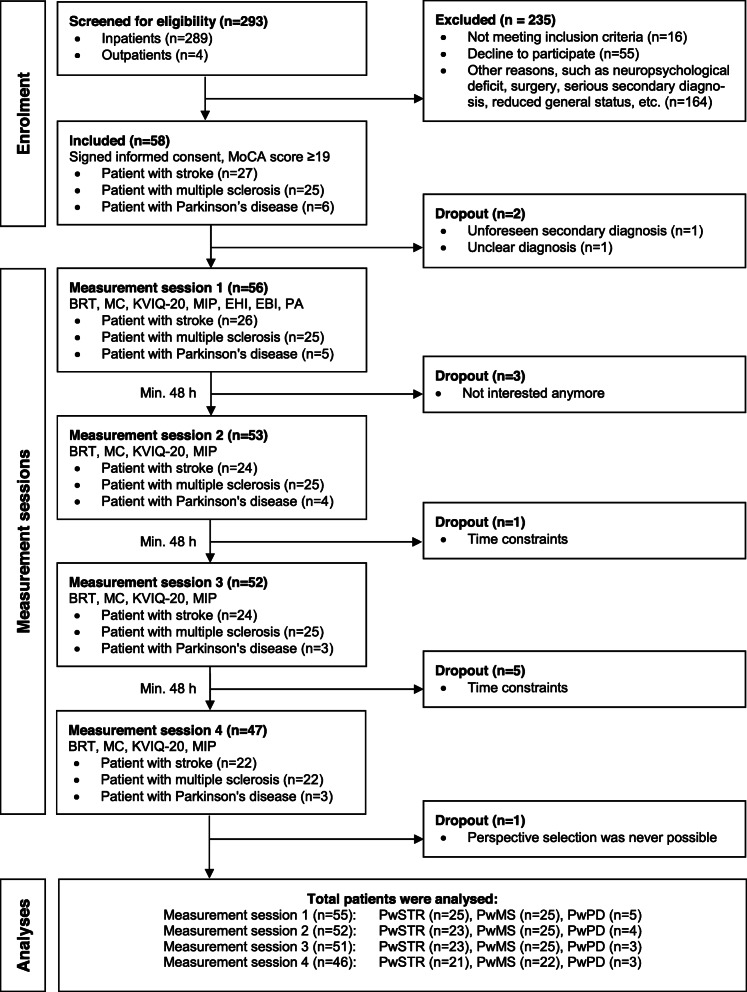
Patient flow chart. Legend: MoCA = Montreal Cognitive Assessment, BRT = Body Rotation Task, MC = Mental Chronometry, KVIQ-20 = Kinaesthetic and Visual Imagery Questionnaire-20, MIP = motor imagery perspective evaluation, EHI = Edinburg Handedness Inventory, EBI = Extended Barthel Index, PA = questions about physical activity, PwSTR = patient with stroke, PwMS = patient with multiple sclerosis, PwPD = patient with Parkinson’s disease, n = sample size, Min= minimum, h = hours

A total of 58 patients were enrolled in the study and data from 55 patients could be analysed. Figure 1 presents the patient flow chart and reasons for drop out. Descriptive parameters for patients’ personal data and assessment scores are provided in Table 1.

**Table 1:** Patients’ characteristics

Group	Stroke *n* = 25	Multiple Slerosis *n* = 25	Parkinson’s Disease *n* = 5	Total *n* = 55
Age (years)	63.3 ± 13.5 (42–91)	51.0 ± 11.9 (24–78)	70.4 ± 3.3 (66–75)	58 ± 14 (24–91)
Gender (female/male)	9/16	16/9	0/5	25/30
Experience with MI (yes/no)	6/19	5/20	0/5	11/44
Time since disease onset (months)	13.6 ± 35.3 (0.6–147.4)	156.0 ± 138.9 (0.7–573.2)	143.3 ± 99.0 (22.5–272.4)	90.1 ± 121.8 (0.6–573.2)
Handedness before diagnosis (right/left)	24/1	25/0	5/0	54/1
^b^National Institutes of Health Stroke Scale (max. 42) *n* = 11	3^a^ (0–14)	n.a.	n.a.	n.a.
^b^Expanded Disability Status Scale (max. 10) *n* = 23	n.a.	6^a^ (2.0–7.5)	n.a.	n.a.
^b^Hoen and Yahr Scale (max. 5) *n* = 4	n.a.	n.a.	3.5^a^ (3–4)	n.a.
Affected body side (right/left/both)	11/14/0	8/14/3	1/4/0	20/32/3
Extented Barthel Index (max. 64)	58.6 ± 6.8 (41–64)	57.3 ± 8.0 (36–64)	60.8 ± 4.3 (54–64)	58.2 ± 7.2 (36–64)
Activity level present (inactive/ partially active/ active)	4/8/13	0/18/7	2/1/2	6/27/22
Activity level past (inactive/ partially active/ active)	0/3/22	0/8/17	0/1/4	0/12/43
Montreal Cognitive Assessment (max. 30)	24.7 ± 2.6 (19–29)	25.4 ± 2.1 (20–30)	22.6 ± 2.9 (19–27)	24.9 ± 2.5 (19–30)

Numbers are listed as frequency or median^a^ or mean score ± SD (range). ^b^Value describing disease status/ quantifying disability caused by the disease. Activity level ‘present’ considered the last 12 months, activity level ‘past’ considered the time before the last 12 months

*KVIQ-20* Kinaesthetic and Visual Imagery Questionnaire-20, *MI* Motor imagery, *n* Sample size, *n.a.* Not applicable, *max.* Maximum score

### Materials and procedure

To evaluate the spontaneously selected perspective, a longitudinal study was conducted. The four measurement sessions were scheduled within 2 weeks at least 48 hours apart to minimise the memory effect of the previous session [38]. One of three trained physiotherapists carried out the standardised measurement procedure. Patients personal and diagnosis-related characteristics were collected from the patients’ medical report and from the patients directly at the beginning of the first measurement session. Furthermore, patients were asked if they had previous experience with MI. Patients’ cognitive function, handedness, and level of independence in activities of daily living were determined using validated instruments, and information about their physical activity level were gathered at the first measurement session. Only MI ability and their preferred perspective were assessed at all four measurement sessions.

#### Cognitive function

Cognitive ability was tested with the MoCA. That instrument screens for mild cognitive dysfunction in different cognitive domains [39]. The lowest possible score is zero, the highest is 30. As the included population often presents mild cognitive impairment, the cut-off for inclusion was set at 19 [40]. That scoring has been shown to have the highest diagnostic accuracy to discriminate between mild cognitive impairment and Alzheimer’s disease (sensitivity 77%, specificity 80%) [40].

#### Handedness

The patients’ dominant side was identified with the Edinburgh Handedness Inventory (EHI) [41]. Patients were assessed by self-reported hand-use for 10 daily activities and the use of eyes and feet in one activity each.

#### Level of independence in activities of daily living

The Extended Barthel Index (EBI) is a reliable (0.96–0.99) and valid tool to rate the level of independence in daily life [42]. The EBI includes 16 items representing different daily activities and cognitive ability (e.g. problem solving). Each item can be rated from zero (fully dependent) to four (fully independent). The total score can reach 64, with a higher score representing a higher level of independence.

#### Physical activity level

Physical activity level categories were defined based on the combination of recommendation from the Federal Office of Statistics - Swiss Health Survey and the handbook for Physical Activity Promotion in Primary Care [43, 44] as follows: inactive (not a noteworthy activity), partially active (at least 30 min per week at a moderate intensity or 1 day per week with vigorous activity resulting in sweating), active (at least 150 min at moderate intensity or 75 min with vigorous activity per week). Patients were asked about all kinds of physical activity including leisure or sport activities, physical activity during work, household, and mobility. They reported the frequency and volume of physical activity twice: for (a) ‘present’ (the last 12 months), and for (b) ‘past’ (the years before the last 12 months), and were assigned to a physical activity level category as described above.

#### Motor imagery ability assessments

The individual MI profiles were assessed using the Body Rotation Task (BRT), Mental Chronometry (MC), and the German version of the Kinaesthetic and Visual Imagery Questionnaire-20 (KVIQ-20). Each of these assessments address distinct MI components [45] and should be combined when assessing MI ability [46]. BRT and MC were carried out in randomised order to reduce a learning effect.

- The BRT is a cognitive task in which individuals imagine moving their body parts from their actual posture into that of the presented position [47]. The assessment focuses on accuracy referring to the number of correct answers. A total of 32 pictures of hands and feet (Recognise Flash Cards, Neuro Orthopaedic Institute, Adelaide City West, Australia) were presented in four different perspectives (back, palm or sole, radial and ulnar, or medial and lateral side) in four various rotations (0, 90, 180, and 270 degrees) on a computer screen in a randomised order. Patients had to decide if the pictured hand or foot was shown from the right or left body side. The pictures remained on the screen until patients responded, but for a maximum of 10 seconds each.
- MC is a reliable method to examine the temporal structure of MI [48]. It measures the temporal congruency between imagining and executing a movement. In the present study, patients performed a grasping and drinking movement with a plastic cup [49]. The examiner demonstrated the task once and the patient was then allowed to practice the task physically and mentally until the examiner could confirm that the patient was able to comply and understood the procedure. For both conditions, the movement began after the examiner gave a start command (‘go’). The end of the movement was defined as moment when the cup first touches the table. For the MI condition, the end of the movement was indicated by the patient with a knock on the table or saying ‘stop’. The examiner manually timed the task with a stopwatch. The trial was repeated three times and average values across all three trials were calculated. To determine MC, the ratio of the time needed for the imagery and physical condition was calculated.
- The KVIQ-20 is valid and reliable questionnaire developed for the MI assessment in patients with sensorimotor impairments [23, 29]. The aim of the KVIQ-20 is to determine the MI ability to visualise and feel the imagined movements using a five-point rating scale (1 = no image to 5 = image as clear as seeing, respectively 1 = no sensation to 5 = as intense as executing the movement). The KVIQ-20 includes 20 items (10 movements in each of visual and kinaesthetic subscales) representing simple movements of the head, shoulders, trunk, upper limbs, and lower limbs in a sitting position (Table 2). Upper and lower limb movements were evaluated on both body sides. Altogether, 17 movements were performed on each subscale with a possible scoring ranging from 17 (lowest) to 85 (highest). The standardised instructions and procedures contained four steps: (a) demonstration of the task by the examiner, (b) execution of the task by the patient with assistance from the examiner if necessary, (c) imagination of the task, and (d) rating of vividness or sensation by the patient. To allow the patients the spontaneous selection of their preferred perspective, the instruction of the KVIQ-20 to imagine the movements from the internal perspective was omitted.

**Table 2:** Items of the Kinaesthetic and Visual Imagery Questionnaire-20 and their movement type categories

Visual	Kinaesthetic	Movements	Movement type
1V	1K	Bend and stretch the neck	Axial
2V	2K	Shoulder shrugging	Axial
3Vnd	3Knd	Lift arm forward completely on the non-dominant body side	Upper limb, Proximal
4Vd	4Kd	Bend and stretch elbow on the dominant body side	Upper limb, Distal
5Vd	5Kd	Thumb to fingertips on the dominant-body side	Upper limb, Distal
3Vd	3Kd	Lift arm forward completely on the dominant body side	Upper limb, Proximal
4Vnd	4Knd	Bend and stretch elbow on the non-dominant body side	Upper limb, Distal
5Vnd	5Knd	Thumb to fingertips on the non-dominant body side	Upper limb, Distal
6V	6K	Bend the trunk forward	Axial
7Vnd	7Knd	Stretch out the knee on the non-dominant body side	Lower limb, Distal
8Vd	8Kd	Move the leg to the side on the dominant body side	Lower limb, Proximal
9Vnd	9Knd	Foot tapping on the non-dominant body side	Lower limb, Distal
10Vd	10Kd	Turn the foot outwards on the dominant body side	Lower limb, Distal
7Vd	7Kd	Stretch out the knee on the dominant body side	Lower limb, Distal
8Vnd	8Knd	Move the leg to the side on the non-dominant body side	Lower limb, Proximal
9Vd	9Kd	Foot tapping on the dominant body side	Lower limb, Distal
10Vnd	10Knd	Turn the foot outwards on the non-dominant body side	Lower limb, Distal

*V* Visual subscale, *K* Kinaesthetic subscale, *d* Dominant, *nd* Non-dominant

#### Motor imagery perspective selection

**Fig. 2:**
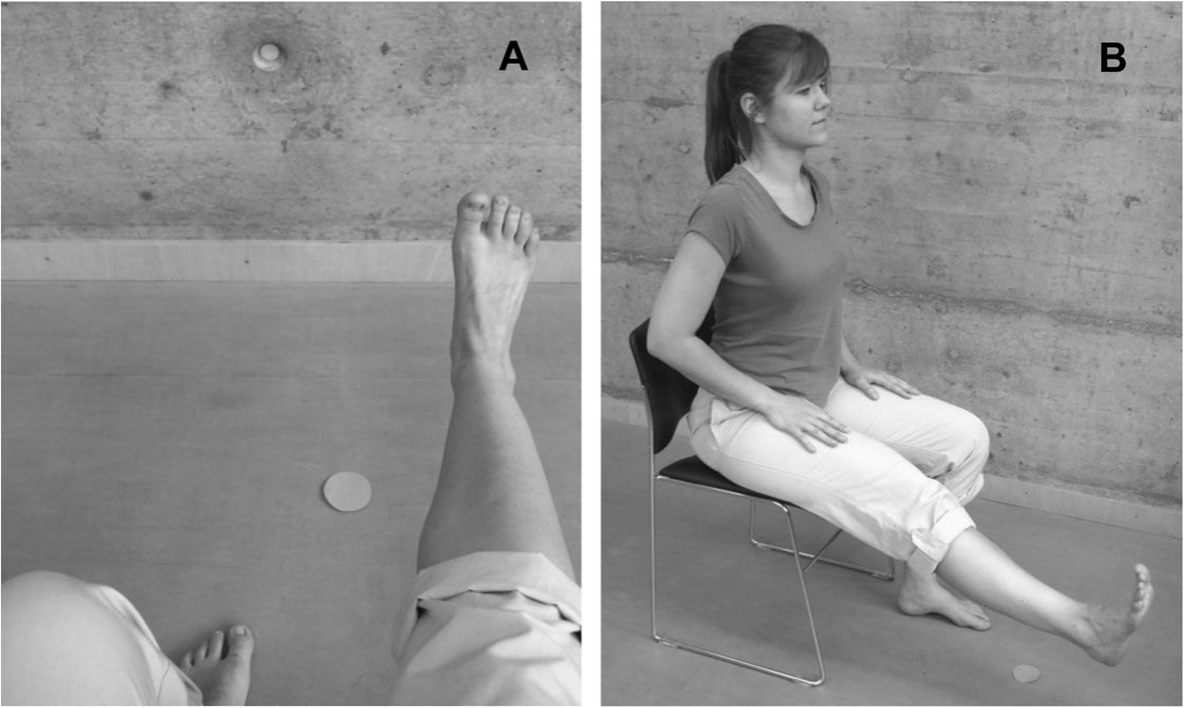
**A** and **B** Motor imagery perspectives: **A** internal, **B** external

The KVIQ-20 was extended with the evaluation of the preferred perspective. Two photographs of each item of the KVIQ-20 were shown to the patients: one photograph representing the internal and one representing the external perspective. After each KVIQ-20 item, patients were asked what photograph represented their preferred perspective. Examples of photographs representing the perspectives are presented in Fig. 2A-B. If a patient changed his/her perspective during imaging a movement of the KVIQ-20 the answer ‘both’ was noted down. In case a patient could not decide on one perspective, the answer ‘perspective selection not possible’ was noted down. All items of KVIQ-20 were evaluated as above described first for the visual and subsequently for the kinaesthetic subscale.

### Data analyses

The Statistical Package for the Social Sciences (Version 23.0 SPSS IBM, Armonk, USA), Excel (Version 2011 for Mac, Microsoft Corporation, Redmond, USA), and the statistical software R (4.02) were used for analyses. Descriptive data were calculated including frequencies, mean and standard deviation, median and range where appropriate for patients’ personal and assessment data. Normality assumption of the MI ability scores (KVIQ-20, BRT, MC) measured at four measurement sessions were tested graphically using QQ-Plot and analysed using the Friedman Test.

Patients (age range 24–91) were assigned to three age groups: < 45 years (adult), 45–64 years (middle aged), and > 64 years (aged) based on the Medline MeSH classification of age categories [50]. A cut-off at 64 years was set due to the findings of Mulder et al. [31].

Frequency analyses on both visual and kinaesthetic subscales were conducted considering every single perspective selection across all KVIQ-20 items for the whole study population.

The frequencies of overall changes in the MI perspective and its consistency were analysed on the data from all four measurement sessions as follows:

- The frequency of changes in one KVIQ-20 movement was analysed. There could be a maximum of three and a minimum of no changes over all four measurement sessions for one out of 17 KVIQ-20 movements per patient. For all KVIQ-20 movements there could be a maximum of 51 changes per subscale (102 for both subscales) and a minimum of zero. These changes were calculated and compared for each measurement session.
- Based on the patients’ number of changes they were assigned to five categories: (1) no changes, (2) few changes: one to 12 changes, (3) moderate changes: 13 to 25 changes, (4) frequent changes: 26 to 38 changes, and (5) very frequent changes: 39 to 51 changes based on cut-off percentages for the categories at 25, 50, 75% of all possible changes. Additionally, descriptive data were listed for patients of each change-behaviour category.

The KVIQ-20 items were subdivided into movement groups: axial, upper limb, lower limb, dominant side, non-dominant side, proximal, and distal (Table 2). The frequency analysis of selected perspectives on both visual and kinaesthetic subscales were performed for all movement groups by calculating the numbers of patients, who preferred the internal or external perspective or whose perspective selection was not constant. An internal perspective preference was considered if a patient used an internal perspective during imaging of every item of a movement group. Likewise, an external perspective preference was determined if a patient used an external perspective during imaging of every item of a movement group. If a patients’ preferred perspective altered at least once during imaging every item of a movement group, e.g. a patient imagined two out of three items of the axial movements using an external MI perspective, however, by imaging the remaining item, an internal perspective was preferred, an altered perspective was determined.

#### Statistical model description

To investigate the relationship between the MI perspective preference and the explanatory variables age, activity levels (present (AL1), past (AL0)), movement type (type), imagery modality (mode) and the different assessments (KVIQ, BRT, MC), the following logistic mixed-effects model with crossed random effects was used:

$$ {\displaystyle \begin{array}{c} logit\left({\pi}_{itk}\right)={\beta}_0+{\beta}_1\  ag{e}_i+{\beta}_2\  AL{0}_i+{\beta}_3\  AL{1}_i+{\beta}_4\  typ{e}_i+{\beta}_5\ \mathit{\operatorname{mod}}{e}_i\\ {}+{\beta}_6\  KVI{Q}_i+{\beta}_7\ B{RT}_i+{\beta}_8\ M{C}_i+{\beta}_9\  time\ poin{t}_i\\ {}+{u}_i+{v}_t,\end{array}} $$

whereas *π* models the probability that the patient uses an internal perspective. The variables *u*, *v* defined the random effects for the patient and the time point respectively. This logistic random-effects model adequately addressed the dependency structure within the data, i.e. the numerous repetitions *k* per patient *i* on four different time points *t*.

## Results

### Patients’ motor imagery assessment scores

A nonparametric test was used for analysing MI assessment scores over four measurement sessions as the assumption of normal distribution was violated. The results of the Friedman Test upon MI ability assessments scores are reported in Table 3. Measurement session was used as an independent variable in the analyses. There was no significant change in the visual subscale of the KVIQ-20 (Χ^2^(3) = 4.453, *p* = 0.217) and in the MC (Χ^2^(3) = 3.190, *p* = 0.363) over the four measurement session. The score in the kinaesthetic subscale of the KVIQ-20 increased significantly (Χ^2^(3) = 13.204, *p* = 0.004), as well as the score in the BRT (Χ^2^(3) = 13.911, *p* = 0.003).

**Table 3:** Patients’ motor imagery ability assessments scores over four measurement sessions

*n* = 46	ME 1	ME 2	ME 3	ME 4	Χ^2^(3)	*P*-value^a^
KVIQ-20 visual subscale (max. 85)	63.5 (24)	64.5 (25)	62.5 (26.25)	64.0 (29.25)	4.453	0.217
KVIQ-20 kinaesthetic subscale (max. 85)	57.0 (19.5)	58.5 (25.25)	61.0 (28)	64.5 (23.75)	13.204	0.004
Body Rotation Task (max. 32)	28.0 (5.25)	26.5 (8)	28.0 (6.25)	28.0 (8.25)	13.911	0.003
Mental Chronometry Quotient (imagery/ physical execution)	1.01 (0.38)	1.04 (0.25)	0.99 (0.23)	0.95 (0.21)	3.190	0.363

Numbers are listed as median (interquartile range)

*n* Sample size, *ME* Measurement session, *KVIQ-20* Kinaesthetic and Visual Imagery Questionnaire-20

^a^Friedman test

### Frequency analyses of patients’ motor imagery perspectives preference

**Fig. 3:**
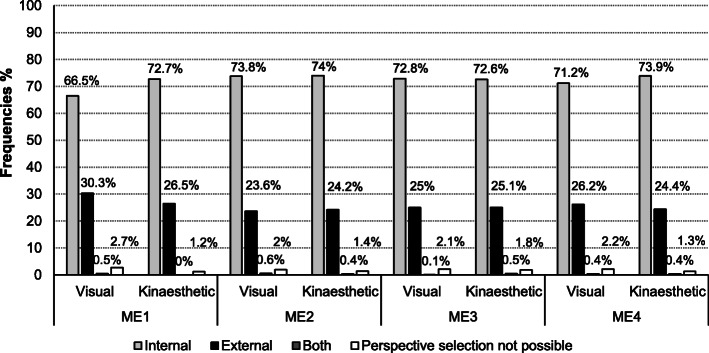
Frequencies of MI perspective preference over all measurement sessions. Legend: ME = measurement session

The frequency analyses of the MI perspective selection for the visual and kinaesthetic subscales at each measurement session are shown in Fig. 3. In general, the internal and external perspectives were selected over all four measurement sessions. In rare cases, patients were not able to select one of the MI perspectives. On the **visual subscale** the external perspective was used in one third of the perspective selection (30.3%) at the first measurement session, which was reduced to one quarter of all selections at the three following measurement sessions (23.6–26.2%). On the **kinaesthetic subscale** the internal perspective was more frequently selected (72.7–74%) over all four measurement sessions compared to the visual subscale. Spontaneous selections of preferred perspectives per movement group on the visual and kinaesthetic subscales assessed at the first measurement session are presented in Table 4.

**Table 4:** Frequency analysis of MI perspective selection in the KVIQ-20 according to movement types

***n*** = 55	Internal perspective		External perspective		Altered perspective		
Movement type	Vis	Kin	Vis	Kin		Vis	Kin
**Axial (3 items)**	15	20	15	8	Total	25	27
					2 movements internal perspective	9	13
**Upper limb (6 items)**	23	26	3	1	Total	29	28
					≥ 4 movements internal perspective	15	9
**Lower limb (8 items)**	22	28	2	4	Total	31	23
					≥ 6 movements internal perspective	12	8
**Dominant side (7 items)**	20	26	1	0	Total	34	29
					≥ 5 movements internal perspective	13	9
**Non-dominant side (7 items)**	22	27	2	0	Total	31	28
					≥ 5 movements internal perspective	9	8
**Proximal (4 items)**	20	26	5	5	Total	30	24
					3 movements internal perspective	12	8
**Distal (10 items)**	21	26	1	0	Total	33	29
					≥ 7 movements internal perspective	14	9
**All (17 items)**	10	20	1	0	Total	44	35
					≥ 13 movements internal perspective	15	9

Numbers in the columns represent the amount of patients, who chose a perspective (out of a total of 55 patients) during visual (Vis) and kinaesthetic (Kin) imagery

In the column of an altered perspective the second row represents 75% of the movements in the movement group and amount of patients, who used an internal perspective at least in 75% of the movements

*KVIQ-20* Kinaesthetic and visual imagery questionnaire-20, *n* Total number of patients

**Fig. 4:**
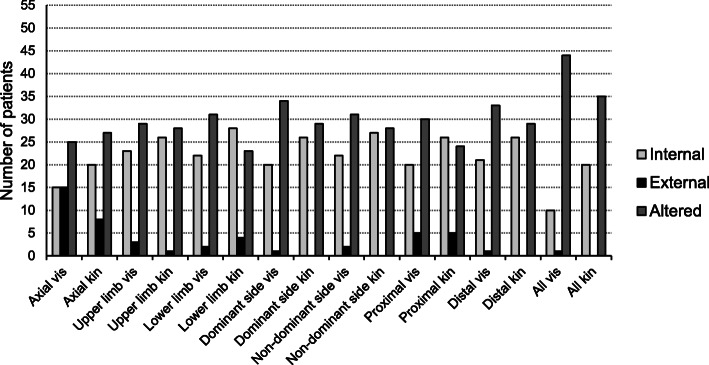
Comparison of MI perspective preferences in different movement type categories of the KVIQ-20. Legend: vis = visual, kin = kinaesthetic, KVIQ-20 = Kinaesthetic and Visual Imagery Questionnaire-20

During kinaesthetic imagery, twice as much patients used an internal perspective for all the KVIQ-20 movements (20 out of 55) compared to visual imagery (10 out of 55) Only one patient imagined all visual imagery movements from an external MI perspective. The majority of the patients (80% during visual imagery, 64% during kinaesthetic imagery) altered their perspective during the KVIQ-20 performance. Most patients used an internal perspective for more than 75% of the KVIQ-20 movements (Table 4). However, in individual cases, patients chose the external perspective more frequently than the internal perspective, e.g. 13 out of 17 movements were imagined from external and only four movements from internal MI perspective, or the choice of the MI perspective was evenly distributed. Figure 4 illustrates the MI perspective selection for different movement types.

#### Motor imagery perspective changes

**Fig. 5:**
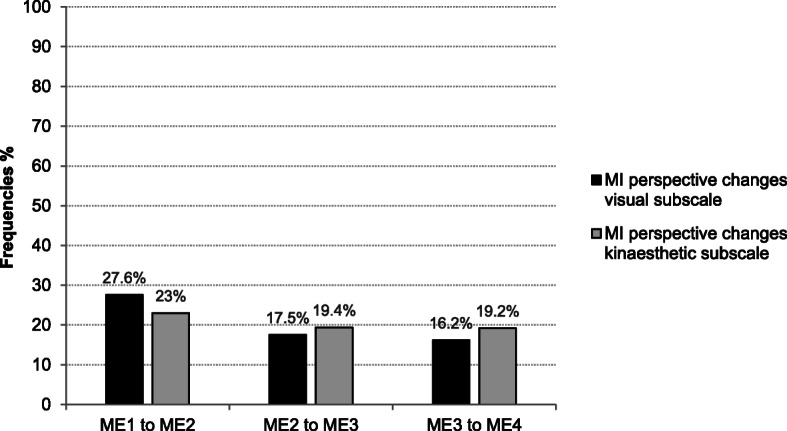
Frequencies of MI perspective changes. Legend: ME = measurement session

There seems to be a slight tendency for selecting an internal perspective over the measurement sessions. Changes of the preferred perspective including preferences for each of the 17 KVIQ items for the visual and kinaesthetic subscale are displayed in percentages in Fig. 5 for each measurement session. In general, more changes of MI perspective selection were observed in the visual subscale (measurement session 1 to measurement session 2: in 27.6% of the imagined items preferred perspective was changed) than in the kinaesthetic subscale (measurement session 1 to measurement session 2: in 23% of the imagined items preferred perspective was changed). The perspective preference became more stable over the third and fourth measurement session.

Patients changed their preferred perspective mainly for four items: lift arm forward completely, bend and stretch elbow, shoulder shrugging, bend and stretch the neck. Patients showed fewer changes for distal upper and lower limb movements: turn the foot outwards, foot tapping, moving thumb to fingertips. Additional file 1 provides detailed information about perspective changes considering the KVIQ-20 movements over the four measurement sessions.

Table 5 provides an overview on the changing behaviour of all patients for the visual and kinaesthetic KVIQ-20 subscales.

**Table 5:** Number of patients in the five categories of change-behaviour

	Category	Number of changes	Visual subscale	Kinaesthetic subscale	Number of stable patients^a^
1	No changes	0	8	13	7
2	Few changes	1–12	24	20	16
3	Moderate changes	13–25	17	15	12
4	Frequent changes	26–38	3	4	2
5	Very frequent changes	39–51	0	0	0
		Total	52	52	37

^a^Patients belong to the same change-behaviour category over all four measurement sessions

In general, patients tended to remain constant regarding their perspective selection for both the kinaesthetic subscale, 13 patients (no changes) and 20 patients (few changes) of the KVIQ- 20 and the visual subscale with eight (no changes) and 24 patients (few changes), respectively. Furthermore, no patient was categorised with very frequent changes*.* Seven patients stayed constant and did not change their perspective selection at all. Additional file 2 provides more information about patients’ characteristics in the different categories of change-behaviour.

#### Age groups and motor imagery perspective preference

Patients’ characteristics in the three age groups are displayed in Table 6.

**Table 6:** Patients’ characteristics in the three age-groups

	Age group < 45 years *n* = 11	Age group 45–64 years *n* = 23	Age group > 64 years *n* = 21
Gender	6 females	11 females	8 females
Mean age	40 ± 5.7	58 ± 13.5	73 ± 6.8.
Diagnosis	2 PwSTR, 9 PwMS	11 PwSTR, 12 PwMS	12 PwSTR, 4 PwMS, 5 PwPD
KVIQ-20 visual subscale	63 (23)	61 (24.5)	66 (17.5)
KVIQ-20 kinaesthetic subscale	57 (8)	57 (19.5)	55 (26)

Numbers are listed as mean ± SD or median (interquartile range)

*KVIQ-20* Kinaesthetic and Visual Imagery Questionnaire-20, *PwSTR* Patients with stroke, *PwMS* Patients with multiple sclerosis, *PwPD* Patients with Parkinson’s disease

**Fig. 6:**
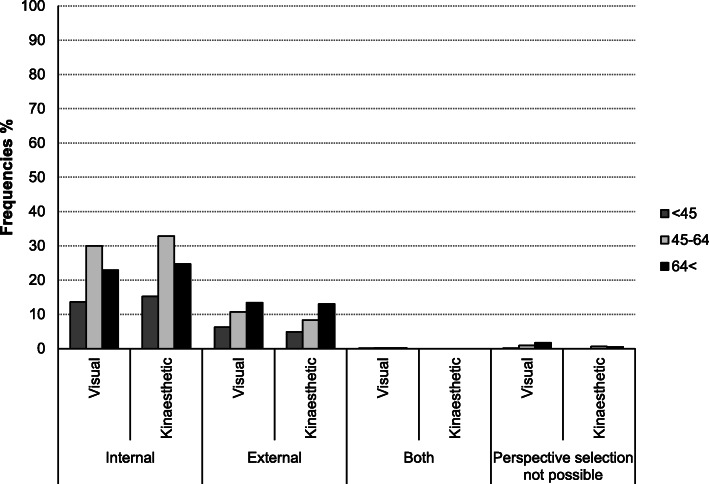
Comparison of MI perspective preferences in three age groups

A frequency analysis showed that patients in the age group > 64 years used an external perspective more frequently and the internal perspective less frequently during visual and kinaesthetic imagery compared to the younger age groups (Fig. 6).

#### Physical activity level and motor imagery perspective preference

**Fig. 7:**
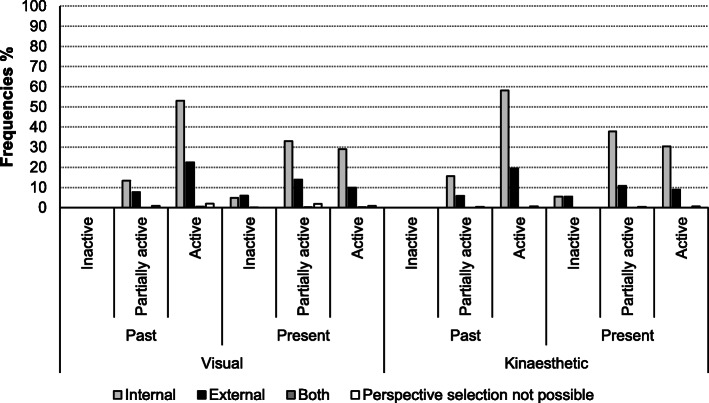
MI perspectives preference in relation to present and past physical activity level of the patients. Legend: activity level present = the last 12 months, activity level past = time before the last 12 months

Figure 7 provides an overview on the perspective preference in the activity level groups. The more active a patient was classified, the more likely he/she used an internal perspective.

### Relationship between motor imagery perspective preference and explanatory variables

The results of the logistic random-effects model are shown in Table 7. The logistic model models the probability to select an internal imagery perspective. The logistic regression estimates give the change in the log-odds of the outcome for a one unit increase in the explanatory variable, e.g. for every one unit increase in age, the log-odds of selecting an internal perspective (versus an external perspective) increases by 0.013. The conditional R- squared of the mixed-effect logistic regression model is 0.65. It describes the amount of variance in the choice of perspective explained by the complete model, i.e. including both fixed and random effects [51]. This seems to be a rather good value considering that a MI perspective is hard to predict.

**Table 7:** The results of the logistic random-effects model

Fixed Effect	Estimate (log-odds ratio)	Std. Error	***P***-value
Imagery modality (visual vs. kinaesthetic)	− 0.0401	0.0782	0.6084
Age	0.0130	0.0251	0.6044
Body Rotation Task score	0.0368	0.0203	0.0698
Mental Chronometry Quotient	−0.2280	0.2247	0.3102
KVIQ-20 score	−0.0140	0.0053	0.0079
Time point (measurement sessions)	0.0515	0.0711	0.4686
Movement type (distal vs. axial)	1.7734	0.1123	< 0.0001
Movement type (proximal vs. axial)	0.9224	0.1206	< 0.0001
Activity level (present; partially acitve vs. inactive)	1.7680	1.1371	0.1200
Activity level (present; active vs. inactive)	2.0726	1.1001	0.0596
Activity level (past)	0.4693	0.8688	0.5891

*KVIQ-20* Kinaesthetic and Visual Imagery Questionnaire-20

In summary, MI modality was not a significant predictor, however, movement type was a statistically significant predictor. Patients were more likely to choose the external MI perspective for axial movements. Time point was not a significant predictor, i.e. patients did not significantly change their MI perspective preference over the four measurement sessions, thus the tendency observed in the frequency analysis could not be confirmed. Furthermore, the KVIQ-20 scores were significant predictors. For an increasing KVIQ-20 score, the probability to select an internal perspective decreased. Age was not a significant predictor, and the ‘present’ physical activity level of a patient was borderline not a significant predictor.

## Discussion

The present study aimed to describe the spontaneous MI perspective selection over four measurement sessions in 55 patients with sensorimotor impairments. Results showed that patients selected both internal and external MI perspectives spontaneously to a large extent when imagining items of the KVIQ-20. MI modality was not a significant predictor of the MI perspective. A significant effect of the KVIQ-20 score and preferred perspective was observed. The higher the KVIQ-20 score was, the lower the probability was to use an internal perspective. Regarding the movement type, the external perspective was more likely used in axial movement types. Over the four measurement sessions, patients became more consistent in their MI perspective preference. Patients changed their MI perspective mainly for the imagination of shoulder, arm and neck movements.

### Motor imagery perspective preference

The KVIQ-20 does not provide an introduction to the concept of MI perspectives, but prompts users to imagine individual items from an internal perspective. Users with a preference on the external perspective might find it difficult to use the internal one and they score lower on the KVIQ-20, resulting in an underestimated MI ability. In further support of this argument, Butler et al. [52] also suggested to examine the relationship between preferred MI perspective and MI ability for an adequate estimation of patients’ MI capacity. In the present study, patients were allowed to choose their preferred perspective spontaneously. At every measurement session, in at least one quarter of all perspective selections the external perspective was chosen. Based on the high percentage of the external perspective preference in our patients, we recommend not to limit the perspective to the internal perspective during KVIQ-20. We would like to encourage researchers and clinicians to provide patients a short introduction to the concept of MI perspectives and to allow a spontaneous selection of the perspective during the KVIQ- 20. This is in line with previous work by Schuster et al. [29], who recommend a pre-assessment of the patient’s ability to distinguish between both MI perspectives before administering the KVIQ-20. In their study the first indications became obvious that patients might not only select the internal perspective if they are offered the internal and external perspective [29]. Scandloa et al. [53] desribed a deficit in MI ability in patients with spinal cord injury regarding the internal perspective but not for the external perspective. In addition, Dettmers et al. [28] reported that patients after stroke showed better MI vividness for the external perspective than for the internal perspective. Jiang et al. [16] emphasized to use different perspectives even according to the location of the brain lesion, so that plasticity or activation of different areas could be supported. For example, it was proposed, that patients with partial brain damage in the temporal lobe should use external imagery to reinforce plasticity in the temporal lobe [16].

Furthermore, the complexity of the imagined movements might play an important role in MI perspective preference. Hardy and Callow [26] suggested that external imagery seems to be a more effective strategy for learning a task that requires the production of complex movement pattern, e.g. the external perspective provides a driver information about different body parts, which would not be possible using an internal perspective. Butler et al. [52] hypothesized that patients after stroke may need to imagine functional movements, e.g. grasping a glass, subdivided in several movement phases. Here, the external perspective might be more efficacious. Scandola et al. [53] desribed a reduced MI ability in patients with spinal cord injury when they imagined a full-body movement compared to an upper extremity movement. Moreover, Kalicinski et al. [32] recommended that the task complexity should be taken into account when MI ability is assessed, as complexity of a task might affect MI performance. A more difficult task leads to a reduced MI ability score [54].

In the present investigation, the majority of the patients altered their preferred MI perspective in the KVIQ-20 during one measurement session. This is similar to what was observed by Schuster et al. [29] and Wondrusch and Schuster-Amft [30]. The authors described, that study patients altered their MI perspective, and that the majority of patients used an external perspective. However, their findings might not be directly comparable with results of the present investigation. The differences to the present study findings might be attributed to the different assessments used to evaluate the MI perspective. Schuster et al. [29] and Wondrusch and Schuster-Amft [30] applied the computer- and video-based Imaprax software [55], which evaluates six complex, multi-joint upper limb gestures or activities of daily living, e.g. to applaud somebody, to write something, compared to the simple movements of KVIQ-20.

In the present study, the high percentage of altered MI perspective selection during one measurement session indicate that further exploration of a preferred MI perspective is profound for a better understanding of the patients’ MI ability.

Altering the MI perspective preference occurred not only within one session. Patients changed their preference between measurement sessions as well. Over four measurement sessions, several changes in the MI perspective selection from one to the next session was observed. Although, the number of changes decreased from the first to the last measurement session, it is difficult to state if patients showed a clear preference to one of both perspective options.

The high number of perspective changes implies that other factors might have an influence on the perspective preference and it might be helpful to evaluate the perspective before an MI training. One influential factor might be the environment where the evaluation setting takes place, e.g. room brightness, auditory properties, setting, or odour. Callow et al. [14] suggested to investigate the relation of other sensory modalities to imagery perspectives such as auditory information. It might influence motor performance as well. In the present investigation, one patient mentioned that he had difficulties getting a clear perspective while sitting in a small room. Another patient mentioned that recruiting the internal perspective was easier when a noise, e.g. foot tapping, or an association with the movement triggered the imagination.

Several authors described that the use of both MI perspectives could be beneficial in different ways depending on the motor task to be imagined and what aspect of the performance should be enhanced [14, 54]. White and Hardy [24] described that imagery from an internal perspective improved motor performance accuracy, thus imagery from an external perspective improved speed. Therefore, changes of MI perspective, or rather the ability to switch between the two MI perspectives might be advantageous and could enhance the accuracy and speed of an imagined task. But these aspects of MI perspectives in motor learning should be further evaluated.

#### Motor imagery perspective preference considering KVIQ-20 items

The association of the preferred perspective to different movement types showed that axial movements of the KVIQ-20 were more frequently imagined using the external perspective. A possible explanation might be that for some movements, the external perspective provides information that would not be gained from an internal MI perspective [26], e.g. during bend head forward. The moving body part itself is not or only partially visible from an internal perspective.

Further results of the present study indicated that the more proximal the executed body movement was the less consistent was the preferred MI perspective for the movement, e.g. lift arm forward completely, bend and stretch elbow, shoulder shrugging, bend and stretch the neck. Moreover, for the imagination of finger and foot movements the least MI perspective changes were observed. A potential reason could be the visibility of the movement or the extent of the body part to be imagined. During small distal movements, e.g. move thumb to finger tips, turning the foot outwards, every moving body part was visible for the patients from the start to the end of the movement. We assume that movement observation before imagination might have made the imagination easier and the same perspective could be selected during each measurement session. In contrast, during the movement lifting arm forward completely, the shoulder could not be seen throughout the complete movement execution. That might have resulted in a less constant perspective selection.

However, in the present study, imaging foot movements remained constant in the MI perspective selection and the least changes occurred over the four measurement sessions. That finding might suggest that prior a MI training the preferred MI perspective of a foot movement should be tested to introduce and explain the perspective.

### Motor imagery perspectives in relation to motor imagery modalities

The findings of the present study did not confirm the association between a kinaesthetic modality and an internal perspective described in the literature [13]. Internal and external perspectives appeared to be independent of the kinaesthetic MI modality, supporting the view that a kinaesthetic sensation is possible during both an internal and an external MI perspective [14, 24].

For future investigations, we would like to encourage researchers to undertake neuroimaging studies with study populations other than stroke and healthy individuals and investigating different modalities and perspectives in relation to age. A precise understanding of what neural structures on which activity level are involved during an internal and an external perspective remains uncertain [14].

### Motor imagery perspective preference and affecting factors

#### Influence of age

Mulder et al. demonstrated a slightly better vividness in healthy elderly people over 64 years when using the external MI perspective [31]. The result of the present study showed that patients with sensorimotor impairments over 64 years preferred to use the external perspective more frequently than younger patient groups. Although this observation was not statistically significant, we consider it to be relevant as it could be an important aspect when using a MI training in neurological rehabilitation.

#### Influence of physical activity level

Mulder et al. described a tendency to use external MI at advanced age based on a lower level of physical activity [31]. Elderly people may be less active and spend more time watching others moving, resulting in a change in their point of view [31]. Based on his propsed hypothesis the association between physical activity level and perspective selection was investigated, independent of age. A borderline not significant effect of physical activity level on perspective selection variables could be shown: the more active patients (at least 150 min at moderate intensity or 75 min with vigorous activity per week) tend to use the internal perspective.

The experience of a certain movement type might be a better predictor of the preferred perspective than physical activity level (volume and frequency). Paris-Alemany et al. [56] recommended to take the familiarity of the movement into account when MI training is introduced as it seems to be decisive for the MI performace. Hardy and Callow suggested that an external perspective might be more beneficial for a task that requires the execution of a complex and form-based movement, e.g. karate [26]. Thus, knowledge about type of activity may be relevant to explain the choice of a particular MI perspective [54].

#### Influence of further aspects

Further variables were suggested to be crucial for the perspective selection. The purpose of a task (e.g. to reach a cup) and the intervention (e.g. early motor skill development) should also be considered [34, 57]. Using a functional, goal-oriented movement instead of the abstract movements of the KVIQ-20 to evaluate perspective might lead to different findings. If a movement is important and familiar to a person it may influence the preferred perspective.

On the other hand, a functional movement may be challenging for patients with motor impairments. A patient may need to imagine phases of a movement separately, or imagine the movement in relation to other body parts [52]. It is hypothesised that the external perspective may be more efficacious for patients in this case [28, 52].

### Study limitations

The nature of MI makes an external control on MI performance difficult. Based on previous research, adding control measures e.g. electro-oculography or recording autonomic nervous system responses might be used as an objective technique to evaluate MI performance [58, 59]. However, these options would provide only information if MI occurred or not, but the MI perspective used and the level of MI vividness would remain uncertain. In the present study, the evaluation of the MI perspective was based on the patients’ verbal description. Most of the patients reported that the photographs used to express MI perspective were helpful and represented well both different perspectives. Additionally, as recommended by McAvinue and Robertson [60], an assessment battery including different assessments, was applied to evaluate different aspects of MI ability.

For the MI perspective selection only movements of the KVIQ-20 were investigated that included single joint movements performed and imagined in a sitting position only. However, for the evaluation of the perspective in patients with sensorimotor impairment is the KVIQ- 20 most suitable because it was specifically developed for that population. Furthermore, the KVIQ-20 included proximal, distal, and axial movements of all body parts.

Finally, the relatively small sample size per patient group did not allow an in-depth examination of correlations among patient group characteristics and perspective preference.

## Conclusions

The present investigation of MI perspectives demonstrated that patients with sensorimotor impairments selected both the internal and external MI perspective spontaneously during visual and kinaesthetic MI over four measurement sessions within 2 weeks. These results indicate that MI modalities and perspectives should be taken into account independently by assessing MI abilities or the effect of MI practice.

Patients with sensorimotor impairments seem to change the MI perspective spontaneously between different visual and kinaesthetic MI tasks. Axial movements were more likely imagined using the external perspective compared to other movement types.

Patients became more consistent in their preferred perspective over time if they imagined finger, hand, and foot movements. However, a statistically significant effect of age on MI perspective preference could not be detected. Patients with a reduced physical activity level showed a decrease in using the internal MI perspective. Foot and finger movements might be selected as an indicator for MI ability. MI perspective controllability over time and its influence on MI practice efficacy should be examined in future investigations.

Our results of the spontaneous MI perspective selection and its changes in a longitudinal observation contributes to a better understanding how patients imagine a movement and how inconsistencies of the MI perspective occur with pursuing measurement sessions. It could be hypothesised that a change of the MI perspective may be advantageous and should be included into a MI training to enhance motor output.

## Supplementary Information

**Additional file 1.** Motor imagery perspective changes considering KVIQ-20 movements.

**Additional file 2.** Patients’ characteristics for each category of change-behaviour.

## Data Availability

The datasets generated and analysed during the current study are available from the corresponding author on reasonable request. All data generated or analysed during this study are included in this published article and its supplementary information files.

## Abbreviations

MI: Motor imagery
KVIQ-20: Kinaesthetic and Visual Imagery Questionnaire-20
fMRI: Functional magnetic resonance imaging
PwSTR: Patients with stroke
PwMS: Patients with multiple sclerosis
PwPD: Patients with Parkinson’s disease
MoCA: Montreal Cognitive Assessment
EHI: Edinburgh Handedness Inventory
EBI: Extended Barthel Index
BRT: Body Rotation Task
MC: Mental Chronometry
V: Visual subscale
K: Kinaesthetic subscale
d: Dominant
h: hours
nd: Non-dominant
min.: Minimum
n: Sample size
PA: Physical activity
n.a.: Not applicable
max.: Maximum score
ME: Measurement session
MIP: Motor imagery perspective evaluation
