# Supplementary material for: A different point of view: the evaluation of motor imagery perspectives in patients with sensorimotor impairments in a longitudinal study
Source: BMC Neurol. 2021 Jul 27;21:297. doi: 10.1186/s12883-021-02266-w (PMC8314460; doi:10.1186/s12883-021-02266-w)
Supplement: Supplementary file 1 — Additional file 1. Motor imagery perspective changes considering KVIQ-20 movements. [file 12883_2021_2266_MOESM1_ESM.pdf]

Additional file 1. MI perspective changes considering KVIQ-20 movements

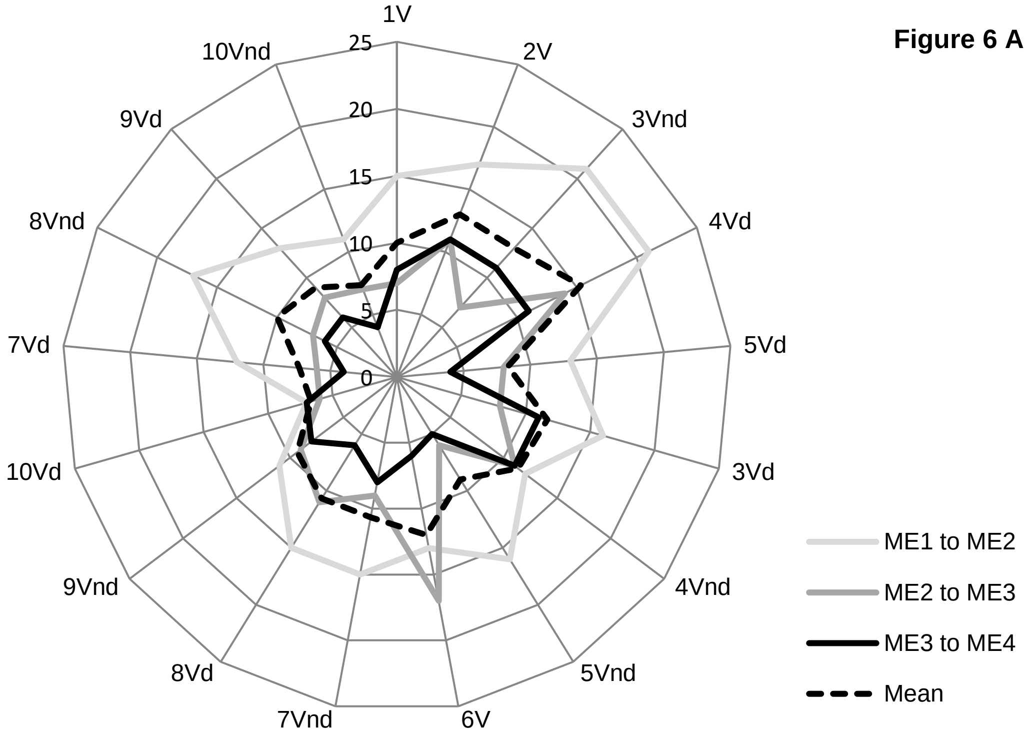

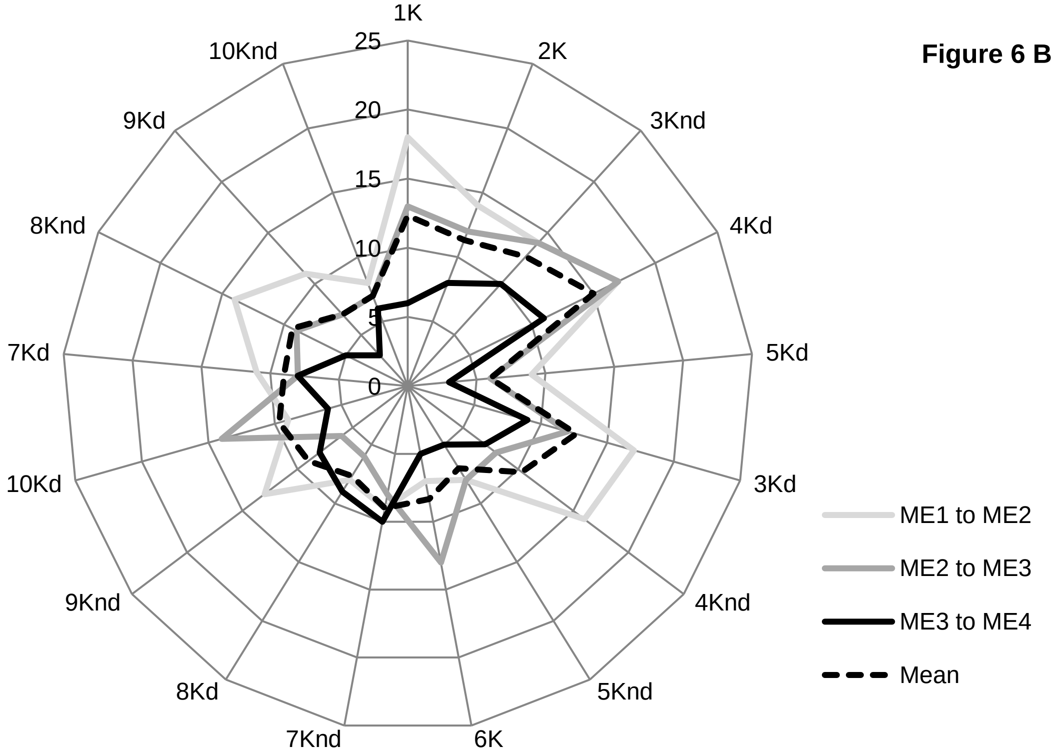

Figure 6: MI perspective changes of all KVIQ-20 items (1-10).

Legend: On visual subscale (6A) and on kinaesthetic subscale (6B) over all measurement sessions. ME=measurement session, Kd=kinaesthetic dominant side, Knd=kinaesthetic non-dominant side, Vd=visual dominant side, Vnd=visual non-dominant side.

Frequent changes in KVIQ-20 movements:

Visual subscale: Patients changed their MI perspective preference from measurement session 1 to measurement session 2 mainly for three items: 3Vnd: lift arm forward completely on non-dominant body side = 21 changes; 4Vd: bend and stretch elbow on dominant body side = 21 changes; and 2V: shoulder shrugging = 17 changes: 59 changes, app. 24.1% of all changes from measurement session 1 to measurement session 2 for the visual subscale.

Kinaesthetic subscale: Patients changed their MI perspective selection from measurement session 1 to measurement session 2 similar to the visual subscale. Changed occurred mainly for item 3Kd: lift arm forward completely on dominant body side = 17; 4Kd: bend and stretch elbow on dominant body side = 17; 1K: bend and stretch the neck = 18; and 2K: shoulder shrugging = 14: 66 changes, app. 33% of all changes from measurement session 1 to measurements session 2 for the kinaesthetic subscale.

Least changes in KVIQ-20 movements:

Visual subscale: Patients showed fewer changes for distal upper and lower limb movements from measurement session 1 to measurement session 2: item 10Vd: turn the foot outwards on dominant body-side: 7 changes = 3% of all changes; 11 changes for item 10Vnd: turn the foot outwards on non-dominant body side; 11 changes for item 9Vnd: foot tapping on non-dominant body side. Each movement accounts for 4.5% of all changes from measurement session 1 to measurement session 2 for the visual subscale. That remained constant or was reduced to 7, 4, and 8 changes from measurement session 3 to measurement session 4.

Kinaesthetic subscale: Item 5Kd/5Knd: moving thumb to fingertips on dominant/non-dominant body side showed the least changes with only 9 respectively 8 changes = 8.4% of all changes between measurement session 1 and 2 for the kinaesthetic subscale.
