# Supplementary material for: A different point of view: the evaluation of motor imagery perspectives in patients with sensorimotor impairments in a longitudinal study
Source: BMC Neurol. 2021 Jul 27;21:297. doi: 10.1186/s12883-021-02266-w (PMC8314460; doi:10.1186/s12883-021-02266-w)
Supplement: Supplementary file 2 — Additional file 2. Patients’ characteristics for each category of change-behaviour. [file 12883_2021_2266_MOESM2_ESM.pdf]

Additional file 2. Patients' characteristics for each category of change-behaviour.

Characteristic	No changes n= 7 (3, 4, 0)	Few changes n= 16 (5, 9, 2)	Moderate changes n= 12 (7, 5, 0)	Frequent changes n= 2 (1, 0, 1)	Very frequent changes n= 0 (0, 0, 0)
Age (years)	56.9±11.6 (39-69)	57±16.5 (24-79)	57.5±12.7 (42-85)	68±9.9 (61-75)	n.a.
Time since disease onset (months)	104.7±158.7 (0.7-369.4)	142±156.7 (0.9-573.6)	69.8±70.2 (0.6-163.2)	47.4±65.8 (0.8-93.9)	n.a.
Extended Barthel Index (max. 64)	61.9±2.7 (58-64)	57.4±7.5 (38-64)	57.5±7 (44-63)	58.5±0.7 (58-59)	n.a.
Montreal Cognitive Assessment (max. 30)	24.1±3.0 (20-27)	24.9±2.5 (19-29)	25.7±1.6 (23-29)	20.5±2.1 (19, 22)	n. a.
KVIQ-20 Vis. subs. (17-85)	71.3±15 (51-85)	60.8±16.5 (25-85)	63.1±16.7 (32-82)	55±10.6 (48-63)	n.a.
KVIQ-20 Kin. subs. (17-85)	59.5±21.4 (22-85)	60.8±13.6 (27-83)	56.6±17.3 (25-80)	52.5±12.4 (44-61)	n.a.
Body Rotation Task (max.32)	29±2.5 (25-32)	28±3.6 (20-32)	27±4 (19-32)	21±3.3 (18-23)	n.a.
Mental Chronometry Ratio	0.94±0.13 (0.72-1.17)	1.03±0.27 (0.82-1.91)	1.07±0.22 (0.8-1.52)	1.03±0.28 (0.49-1.94)	n.a.

Legend: Numbers represent mean±SD (range), n=sample size (in brackets number patients with stroke, with multiple sclerosis, with Parkinson's disease in the category),max.=maximum score, KVIQ-20= Kinaesthetic and Visual Imagery Questionnaire-20, kin. subs.=kinaesthetic subscale, vis. subs.=visual subscale, n.a.=not applicable.

Observed associations between change categories and patient characteristics

Age: A notable association was observed in an increased number of changes with older average age of the patients: 68±9.9; range 61-75.

Time since disease onset: A higher level of MI perspective changes could be observed in patients with less duration of the disease. Average time since disease onset was 47.4±65.8 (range 0.8-93.9) months for category frequent change and 142±156.7 (range 0.9-573.6) months for category few changes.

MI ability: Patients belonging to category frequent changes showed lower scores:

(1) in the KVIQ-20 compared to the other changes categories. The average score was 55±10.6 (range 48-63) on the visual subscale and 52.5±12.4 (range 44-61) on the kinaesthetic subscale.

(2) in BRT: 21±3.3 (range 18-23). In general, it was notable that the more constant patients are in their perspective selection, the higher the scores they achieved on MI ability assessments.
